# The Trade in Oysters

**Published:** 1896-12-12

**Authors:** 


### The Trade in Oysters.

The question of the oyster has been once more brought to the front by the issue of a most careful and admirable report on the whole subject of "Oyster Culture in Relation to Disease" by the Medical Officer to the Local Government Board.

In this report Dr. Thorne Thorne has dealt with the matter in a most judicial spirit. The *British Medical Journal*, in fact, fears that the effect of the report may be to "put back" the question, and says that the chances of the disease germs succumbing in the sewage "are dwelt upon in such detail as to produce the impression that little or no risk is involved in the consumption of such oysters."

But it is this very fairness, that gives to the conclusions arrived at by Dr. Thorne Thorne their great importance.

The accusation against oysters amounts to this: that while millions of them are good and sound, and have been reared and stored in places practically free from all likelihood of contamination, a certain number of oyster "layings" and "storage pits" exist, the surroundings of which are absolutely abominable, and that the consumption of oysters from these sources is full of danger. An oyster properly dealt with is a good, nutritious, and toothsome article of diet, and such are the majority; but mixed with these, and indistinguishable from them by the ordinary consumer, are oysters which have been recklessly exposed to the most disgusting contamination, and are distinctly dangerous.

It is the uncertainty, the impossibility, of knowing whether even the highest priced oyster may not contain a dose of typhoid, that makes wise men eschew such dainties under present conditions of supply, and that involves the oyster trade in such loss as has been complained of, and it seems to us in the highest degree absurd that the dealers in these comestibles, instead of joining in the demand for the suppression of those oyster-breeders who by their bad arrangements are ruining the trade, should vent their anger against those who have shown the danger of eating oysters reared under present conditions.

A great deal of very interesting bacteriological work has been done in investigating this question. It has been shown that the bacillus of typhoid fever can live in sea water for two or more weeks; that oysters collected from certain places which were known to be exposed to contamination by sewage were found to contain certain intestinal microbes which are commonly found in sewage, whereas oysters gathered from places which appeared to be free from risk of sewage contamination did not contain any of these organisms; and lastly, in one sample derived from an especially suspicious source, the actual bacillus of typhoid fever was found in the mingled body and liquor of the oyster.

But it is not on mere laboratory experiments that Dr. Thorne Thorne depends in his indictment of the oyster as a means of conveying typhoid fever. "This fever," he says, "has been ascertained to have followed on the consumption of raw oysters; the interval between such consumption and the onset of the symptoms has corresponded with the incubation-period of the disease; the special incidence of the fever has been on those who were known to have partaken of the oysters, whilst others, who could only be differentiated from the sufferers in that they had not partaken of the oysters, escaped; the consumption of oysters has been ascertained by a process of exclusion to have been the only medium through which such a disease could have been simultaneously communicated to the sufferers; and at times the oysters in question have been found to have had opportunity of contamination by human excreta, even by specifically infected excreta." This is common sense proof by ordinary process of reasoning, and it ought to be enough. The stringency of the proof is, however, enormously increased by the fact that the common sense argument, and the bacteriological argument arrive at the same conclusion, although by entirely independent routes.

We need not go here into the dirty details given in the appendices to the report as to the relation of the oyster beds or "layings" with the sewer outfalls at such places as Southend, Cleethorpes, Grimsby, and in the Medina. But we must express our astonishment that men can be found to object to such regulations as should render such abominations impossible in the future. Here we see discredit thrown on an enormous British industry, supplying a perfectly healthy article of diet, and all for the sake of some half-dozen oyster-growing places in England, and others in foreign parts. We should have expected that the great oyster growers would have been the first to ask for inspection and regulation of the oyster trade. In any case, however, this is what it must come to. The British public wants its oysters and it wants them clean; and the oyster dealers, if they try to stand up against Dr. Thorne Thorne will but find themselves kicking against the pricks.

